# The gut microbiome affects response of treatments in HER2‐negative advanced gastric cancer

**DOI:** 10.1002/ctm2.1312

**Published:** 2023-06-28

**Authors:** Zihan Han, Siyuan Cheng, Die Dai, Yan Kou, Xiaotian Zhang, Fang Li, Xiaochen Yin, Jingjing Ji, Zhikuan Zhang, Xi Wang, Ning Zhu, Qi Zhang, Yan Tan, Xiaohuan Guo, Lin Shen, Zhi Peng

**Affiliations:** ^1^ Department of Gastrointestinal Oncology Key Laboratory of Carcinogenesis and Translational Research (Ministry of Education), Peking University Cancer Hospital and Institute Beijing China; ^2^ Department of Colorectal Surgery China‐Japan Friendship Hospital Beijing China; ^3^ Department of Tumor Chemotherapy and Radiation Sickness Peking University Third Hospital Beijing China; ^4^ Xbiome Shenzhen China; ^5^ Institute for Immunology, School of Medicine Tsinghua University Beijing China

**Keywords:** chemotherapy, gastric cancer, gut microbiome, immunotherapy

## Abstract

**Background:**

Common treatments for metastatic/unresectable HER2‐negative gastric cancer include chemotherapy, immune checkpoint inhibitor monotherapy and chemotherapy plus immune checkpoint inhibitor. However, significant drug resistance exists regardless of the treatment regimen.

**Methods:**

Patients with metastatic/unresectable HER2‐negative gastric/gastroesophageal junction adenocarcinoma were enrolled. All patients were divided into three groups according to the treatment regimen and were further divided into responders and non‐responders according to efficacy evaluation. Metagenomics sequencing were performed to analyze gut microbiome signature of patients receiving different treatments at baseline and throughout treatment.

**Results:**

One hundred seventeen patients with HER2‐negative advanced gastric or gastroesophageal junction adenocarcinoma receiving chemotherapy alone, anti PD‐1/PD‐L1 immunotherapy alone or combined regimen were included in this study. Microbiome signatures related to clinical response are distinct among the three treatment groups. Among which, 14, 8 and 13 species were significantly different between responders and non‐responders in immunotherapy, immunotherapy plus chemotherapy and chemotherapy group, respectively. Patients with higher relative abundance of *Lactobacillus* possessed higher microbiome diversity and significantly better response to anti‐PD‐1/PD‐L1 immunotherapy and had a trend to achieve better progression‐free survival. Another cohort of 101 patients has been used as an external validation set to confirm the stability and reliability of these findings.

**Conclusions:**

Gut microbiome affects response of treatments in HER2‐negative advanced gastric cancer in a treatment‐specific way, immunotherapy plus chemotherapy did not equal to a simple superposition of immunotherapy and chemotherapy. *Lactobacillus* is expected to become a novel choice as an adjuvant agent in promoting the efficacy of immunotherapy in gastric cancer.

## Graphical abstract

Metastatic/unresectable HER2‐negative gastric cancer is a life‐threatening disease and is difficult to cure. This study enrolled 117 gastric cancer patients receiving chemotherapy alone, anti PD‐1/PD‐L1 immunotherapy alone, or combined regimen. Metagenomics analysis was performed to evaluate the impact of gut microbiome on treatment response. We found that microbiome signatures related to clinical response are distinct among the three treatment groups. Patients with higher relative abundance of Lactobacillus possessed higher microbiome diversity and significantly better response to anti‐PD‐1/PD‐L1 immunotherapy and had a trend to achieve better progression‐free survival (PFS).

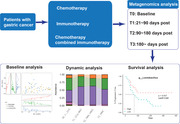

## 1 BACKGROUND

For HER2‐negative advanced gastric or gastroesophageal junction (GEJ) adenocarcinoma, chemotherapies based on platinum,^1^ fluorouracil^2^, ^3^, ^4^ and paclitaxel^5^, ^6^, ^7^ were the most effective treatments in the past decades. However, the therapy has limited effects. With the clinical application of immune checkpoint inhibitors (ICI), the treatment strategies of gastric cancer have been revolutionized. However, the overall response rate (ORR) is only 11%−15%.^8^, ^9^ Only those patients with rare subtypes such as microsatellite instability‐high/deficient mismatch repair (MSI‐H/dMMR^10^, ^11^;) and Epstein‐Barr virus‐associated gastric cancers (EBVaGCs^12^, ^13^;) might benefit from this treatment. This means most patients can hardly benefit from ICI monotherapy. Additionally, how to overcome the ICI resistance is also a critical clinical problem. Chemotherapy plus ICI is a feasible way to partly overcome drug resistance. In CheckMate 649 trial,^14^ combined therapeutic strategy showed an improved clinical benefit in patients with PD‐L1 combined positive scores ≥ 5. Although CheckMate 649 trial^14^ had achieved a positive result, the response and survival rate did not meet expectations. Factors involved in modulating resistance of chemotherapy plus immunotherapy remained unclear.

The interplay between the gut microbiome and immune system plays a pivotal role in cancer patients’ response to immunotherapy and/or chemotherapy. It has been shown in melanoma that the gut microbiome can modify the tumour microenvironment by mediating the infiltration of CD8+ T‐cells,^15^ thereby directly influencing the response to anti‐PD‐1/PD‐L1 immunotherapy. Besides, some studies have shown that the composition of the gut microbiome,^16^ and certain bacterial species in particular (e.g., *Fusobacterium nucleatum* ^17^), can influence the host's response to chemotherapy.

In our previous study, we demonstrated that the ratio of *Prevotella*/*Bacteroides* is associated with ICI resistance in gastrointestinal cancer, whereas this trend is not significant in gastric or GEJ cancer.^18^ Based on this, we further operated a prospective cohort study recruiting patients treated with ICI monotherapy, standard chemotherapy or chemotherapy plus ICI. In order to more accurately reveal the correlation between gut microbiome and the efficacy of treatments of gastric cancer, we first examined the microbial characteristics and differences between responders and non‐responders for each treatment modality and explored the temporal changes of these microbiota. Additionally, we investigated the correlation between these microbiota and progression‐free survival (PFS). Interestingly, we found that *Lactobacillus* was consistently associated with responders across all analyses in both discovery and validation cohort, including differential analysis, temporal analysis, and survival analysis. We further explored the microbial characteristics of individuals with high abundance of *Lactobacillus*.

## 2 METHODS

### 2.1 Cohort recruitment

HER2‐negative advanced gastric or GEJ adenocarcinoma patients were enrolled at Peking University Cancer Hospital. We enrolled 152 patients hospitalized in the Department of Gastroenterology at this hospital between February 2018 and July 2021. To make our results more robust, we have been continuously working to collect the fecal samples from gastric cancer patients. Another cohort of 101 patients (159 samples) with gastric cancer was used as an external validation set. All participants were in stage III/IV per TNM staging (AJCC 8th) and were receiving either chemotherapy (XELOX [Capecitabine 1000 mg/m2 twice daily, days 1−14; oxaliplatin 130 mg/m2, day 1, every 3 weeks]) alone, anti‐PD‐1/PD‐L1 immunotherapy alone or combined treatment. The treatment plan for each patient was decided by experienced physicians at Peking University Cancer Hospital according to treatment guidelines from the National Comprehensive Cancer Network. Because this is a real‐world study with patient recruitment spanning from 2018 to 2021, the guidelines have evolved with slight updates each year. In general, patients with specific molecular characteristics (e.g., MSI‐H, high PD‐L1 expression, EBER‐positive) prefer regimens containing immunotherapy, while others prefer chemotherapy alone. Some treatment options were chosen after adequate physician‐patient communication. Antibiotic usage and clinical examinations were documented for all patients. This study was approved by the Ethics Committee at Peking University Cancer Hospital. Informed consent was obtained from all patients enrolled, and all tests and procedures were conducted in accordance with the Declaration of Helsinki.

Efficacy evaluation was performed in accordance with Response Evaluation Criteria in Solid Tumours version 1.1 (RECIST1.1),^19^ which involves classifying treatment response into four categories: complete response (CR), partial response (PR), stable disease (SD), and progressive disease (PD). CR refers to the disappearance of all target lesions, while PR refers to tumours that have decreased in size. SD refers to tumours that have remained stable in size, and PD refers to tumours that have increased in size or become more widespread. The evaluation was performed every 6 weeks and confirmed no less than 4 weeks from the date first recorded. The classification of patients as responders and non‐responders was based on the current expert consensus from Society for Immunotherapy of Cancer,^20^ which provide a framework for defining and studying resistance to therapy. In particular, the Immune‐Related Response Criteria (irRC) recommends using a duration‐based approach to classify response. Here, we use 6‐month timeframe to define responder and non‐responders. Patients with CR/PR/SD lasting more than 6 months were considered responders, as their condition remained stable for a significant period. In contrast, those patients who were evaluated as PD within 6 months were classified as non‐responders, as their condition progressed within the first 6 months of treatment.

### 2.2 Fecal sample collection and microbiome sample preparation

**FIGURE 1:**
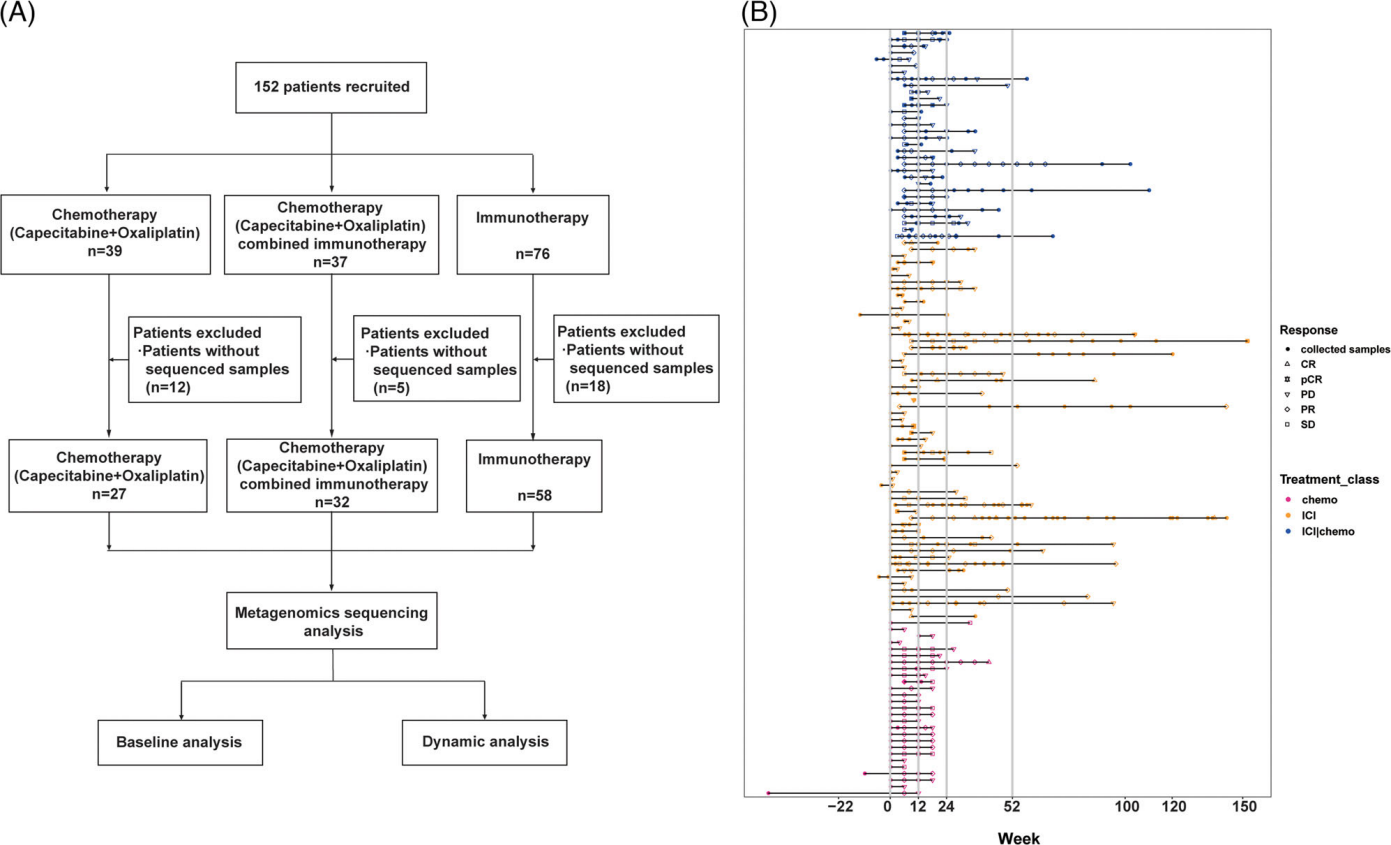
Study workflow and clinical sample collection. (A) One hundred fifty‐two gastric cancer patients were recruited and subdivided into three groups based on treatment. (B) Swimming plot of sample collection and tumour evaluation timeline. The X‐axis shows time as samples were collected and tumour progression was evaluated. The ‘0’ value in X‐axis denotes the time patients began to receive the therapy. Solid dots represent collected samples. Hollow shapes mark evaluated tumour progression. CR, complete response; PD, progressive disease; PR, partial response; SD, stable disease, pCR, pathological complete response.

Stool samples were collected from patients over the course of therapy regimens (Figure 1A), as previously described.^18^ Baseline fecal samples were collected at the outset of treatment, and longitudinal samples were collected continuously throughout the study. All fresh fecal samples were stored at −80°C using Wehealthgene Fecal Microlution Collection kits. Bacterial genomic DNA was extracted using QIAamp PowerFecal DNA Kits (catalog No. 12830−50, Qiagen), according to manufacturer's instructions. DNA extracts were then subjected to library construction and sequencing on an Illumina NovoSeq 6000 platform (Novo Gene).

### 2.3 Metagenomics taxonomic profiling and functional annotation

To obtain high‐quality reads, raw data were quality filtered with KneadData (version 0.6.1), which integrated several QC tools (e.g., Trimmomatic,^21^ FastQC [https://www.bioinformatics.babraham.ac.uk/projects/fastqc/]). Host contaminants were removed by aligning datasets to the human genome (hg19) with Bowtie 2. MetaPhlAn2 (Truong et al., 2015) facilitated taxonomic profiling, and HUMAnN2^22^ coupled with the UniRef90 reference database was applied to functionally annotate metagenomic DNA sequences.

### 2.4 Analysis of metagenomic diversity and differential abundance analysis

Alpha diversity was calculated using the VEGAN R package. Richness and evenness of each sample was then determined. Beta diversity was deduced based on Bray‐Curtis and Jaccard distances, and principal coordinate analyses (PCoA) were applied to identify dissimilarities between groups. To address the complexity of the clinical data, we also examined the influence of confounding clinical variables (e.g., age, sex, tumour stage, prior medication history, body mass index [BMI], MSI status) on microbiome data. We first performed a permutational multivariate analysis of variance (PERMANOVA)^23^ to evaluate the effect of confounding factors on microbial communities. By comparing the between‐group (PERMANOVA P value) and within‐group (beta dispersion P value) differences using PERMANOVA, we can identify whether the observed differences in microbial community composition are driven by true biological factors or by confounding variables. Then we used MaAsLin2^24^ to correct for the confounding factors and identify the gut microbiota most closely associated with response. It offers a correction method called “covariate adjustment,” which aims to remove the effect of confounding variables from the association analysis. This is done by including the confounding variables as additional covariates in the regression model, allowing for a more accurate estimation of the true association between the microbial taxa and the response.

### 2.5 Statistical analyses

Fisher's exact test was applied to estimate associations between clinical characteristics and patient responses. Wilcoxon tests were performed to compare the relative abundance of taxa and pathways between different groups. We employed univariate survival analysis of PFS between different groups via the Kaplan–Meier method and log‐rank tests. *p*‐Values below .05 were considered statistically significant, and all statistical analyses and plotting were conducted in R studio (R version 3.6.3).

## 3 RESULTS

### 3.1 HER2‐negative advanced gastric or GEJ adenocarcinoma cohort

We enrolled 152 patients hospitalized in the Department of Gastroenterology at Peking University Cancer Hospital between February 2018 and July 2021. Of these, 39 received XELOX, 37 received XELOX plus anti‐PD‐L1/PD‐1 immunotherapy, and 76 received anti‐PD‐L1/PD‐1 immunotherapy monotherapy. There were 12, 2 and 18 patients without available sequencing data across these treatment groups, respectively, all of which were omitted from further analyses (Figure 1A). The final cohort consists of 117 patients that were continuously treated and followed up for more than 6 months. This cohort was subdivided into an ICI monotherapy group (*n* = 58), a XELOX plus ICI group (*n* = 32) and a XELOX group (*n* = 27) for all subsequent analyses.

Within each group, patients were further divided into responders (Rs) and non‐responders (NRs), according to their clinical response described above. The ORRs were 37% for the XELOX group, 50% for the XELOX plus ICI group, and 46.5% for the ICI group (Table 1). We first examined the associations between patient characteristics and the response to treatments. The number of patients with MSI‐H subtypes tended to be higher in the response group, though this observation was not statistically significant (*P* = .0548). There were no other statistically significant associations observed between clinical benefits and metadata (Table 2). A total of 279 specimens from 117 patients were subjected to metagenomic sequencing analyses (Figure 1B).

**TABLE 1:** Anti‐tumour activity in patients.

Variable	Immunotherapy (*N* = 58)	Chemotherapy plus immunotherapy(*N* = 32)	Chemotherapy (*N* = 27)
Overall response rate	46.5%	50.0%	37.0%
Response Group			
R	27	11	13
NR	31	21	14

*Note*: Responders were defined as best response is complete response (CR), partial response (PR) or stable disease (SD) and lasts more than 6 months. Non‐responders were defined as best response is progressive disease or CR/PR/SD lasts less than 6 months. Overall response was defined as a confirmed CR or PR.

Abbreviations: NR, non‐responders; R, responders.

**TABLE 2:** Demographic and patient characteristics at baseline.

Characteristics	Immunotherapy (*N* = 58)			Chemotherapy combined immunotherapy(*N* = 32)			Chemotherapy (*N* = 27)		
	R (*N* = 27)	NR (*N* = 31)	Fisher.p	R (*n* = 11)	NR (*n* = 21)	Fisher.p	R (*N* = 13)	NR (*N* = 14)	Fisher.p
**Age**									
Median(range)‐yr	59 (31–80)	58 (27–81)		63 (49–74)	63 (27–72)		62 (51–77)	60.5 (46–71)	
≥65 yr‐no. (%)	12 (44)	10 (32)	.4199	4 (36)	10 (48)	.712	5 (38)	4 (29)	.6946
<65 yr‐no. (%)	15 (56)	21 (68)		7 (64)	11 (52)		8 (62)	10 (71)	
**Sex**									
Male‐no (%)	17 (63)	23 (74)	.4041	9 (82)	18 (86)	1	12 (92)	11 (79)	.5956
Female‐no (%)	10 (37)	8 (26)		2 (18)	3 (14)		1 (8)	3 (21)	
**ECOG performance‐status score‐no (%)**									
0	12 (44)	14 (45)	1	6 (55)	5 (24)	.1228	1 (8)	4 (29)	.3259
1	15 (56)	17 (55)		5 (45)	16 (76)		12 (92)	10 (71)	
**Primary location at diagnosis‐no (%)**									
Gastroesophageal junction	9 (33)	7 (23)	.3929	1 (9)	9 (43)	.1058	7 (54)	4 (29)	.2519
Stomach	18 (67)	24 (77)		10 (91)	12 (57)		6 (46)	10 (71)	
**Histologic subtype‐no. (%)**									
Diffuse	6 (22)	13 (42)	.4168	3 (27)	1 (5)	.211	0	1 (7)	.1962
Intestinal	12 (44)	9 (29)		5 (46)	10 (48)		7 (54)	11 (79)	
Mixed	7 (26)	7 (23)		3 (27)	6 (28)		5 (38)	2 (14)	
Unknown	2 (8)	2 (6)		0	4 (19)		1 (8)	0	
**Previous gastrectomy no. (%)**									
Yes	11 (41)	13 (42)	1	1 (9)	4 (19)	.6367	5 (38)	4 (29)	.6946
No	16 (59)	18 (58)		10 (91)	17 (81)		8 (62)	10 (71)	
**TNM staging**									
III	2 (7)	1 (3)	.5931	0	1 (5)	1	0	0	1
IV	25 (93)	30 (97)		11 (100)	20 (95)		13 (100)	14 (100)	
**PD‐L1 combined positive score‐no. (%)**									
≥5	13 (48)	8 (26)	.1353	4 (33)	4 (19)	.3932	5 (38)	3 (21)	.4989
<5	6 (22)	6 (19)		3 (25)	8 (38)		5 (38)	5 (36)	
NA	8 (30)	17 (55)		5 (42)	9 (43)		3 (24)	6 (43)	
**MSI status‐no. (%)**									
MSI‐high	13 (48)	7 (23)	.0548	2 (18)	2 (9)	.81	0	0	1
Non‐MSI‐high	14 (52)	24 (77)		8 (73)	17 (82)		13 (100)	14 (100)	
NA	0	0		1 (9)	2 (9)		0	0	
**EBER‐no. (%)**									
Positive	6 (22)	9 (29)	.2677	0	1 (5)	1	0	1 (7)	1
Negative	20 (74)	17 (55)		10 (91)	18 (85)		13 (100)	13 (93)	
NA	1 (4)	5 (16)		1 (9)	2 (10)		0	0	
**Prior line of therapy**									
0	11 (41)	8 (26)	.5083	11 (100)	20 (95)	1	13 (100)	13 (93)	1
1	6 (22)	9 (29)		0	1 (5)		0	1 (7)	
2	10 (37)	14 (45)		0	0		0	0	

*Note*: Percentages may not total 100 because of rounding.

Abbreviations: NR, non‐responders; R, responders.

For the validation cohort, we enrolled 101 patients with gastric cancer with detailed demographic and treatment information showing in Table S1. Among the enrolled patients, 48 individuals were treated with ICI, 56 with a combination of ICI and chemotherapy and 2 with chemotherapy only. Total 159 fecal samples were collected and sequenced. This independent cohort has been used as an external validation set to confirm the stability and reliability of our findings.

### 3.2 Microbial composition signatures between three treatment groups

**FIGURE 2:**
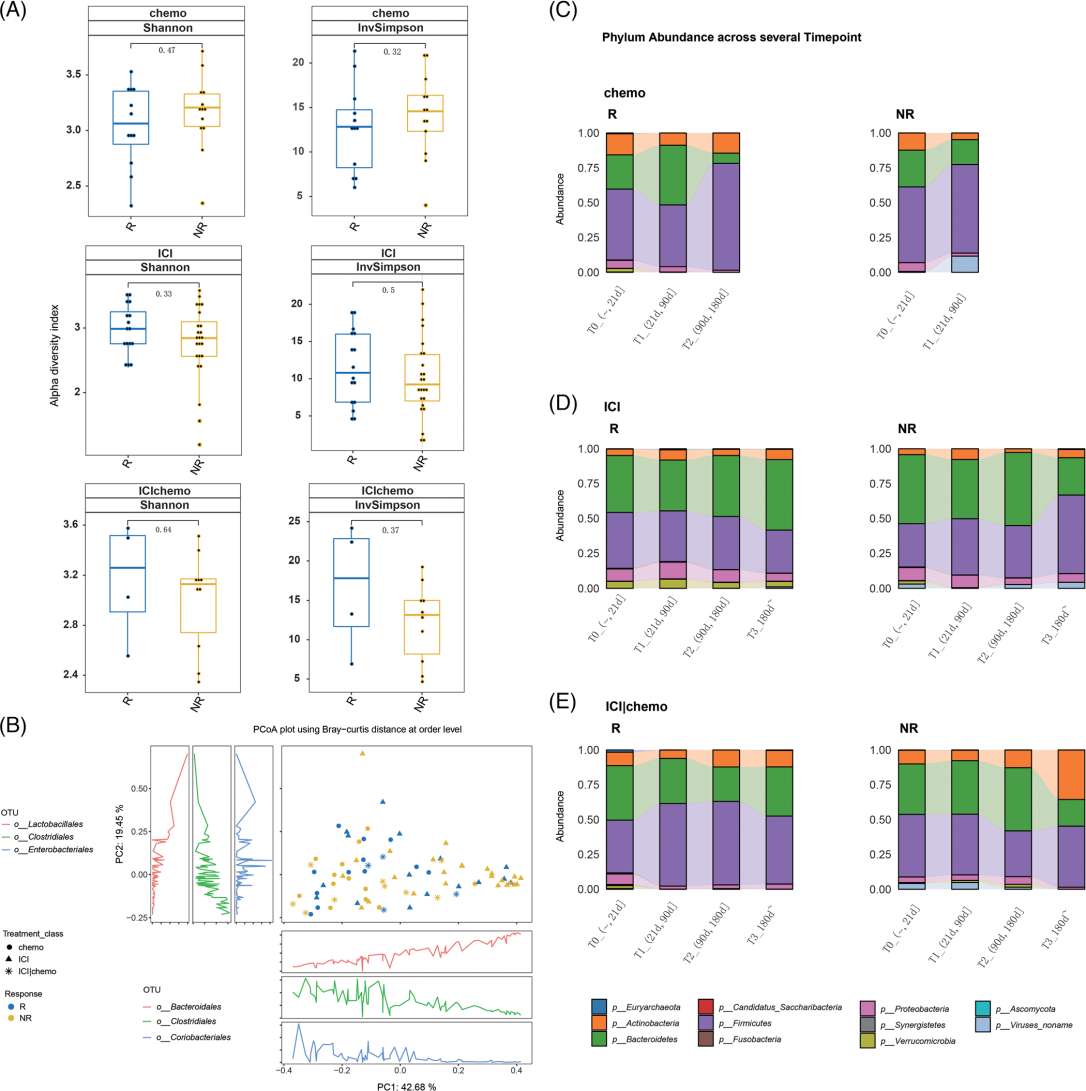
Gut microbiome composition associated with treatment and drug response. (A) Gut microbiome alpha diversity between responders and non‐responders at baseline for the three treatments, respectively (Wilcoxon test). (B) PCoA plot of baseline fecal samples depicts beta diversity evaluated by Bray‐Curtis distance arranged by response and treatment. The x‐ and y‐axes show the first and second principal coordinates, respectively, alongside percentage of variances explained for each dimension. NR, non‐responders; R, responders. (C) Dynamic microbial composition of Rs and NRs in chemotherapy group at the phylum level. (D) Dynamic microbial composition of Rs and NRs in ICI group at the phylum level. (E) Dynamic microbial composition of Rs and NRs in ICI plus chemotherapy group at the phylum level.

We first investigated the gut microbiome composition for all three patient groups. Results showed that the alpha diversity of the gut microbiome at baseline did not differ between Rs and NRs across all three treatment groups (Figure 2A). In addition, the diversity level remains relatively consistent in responders among all three treatments, while in non‐responders, patients receiving ICI alone showed significantly lower baseline diversity comparing to those received chemotherapy alone (Figure S1A, *P* = .026 for Shannon indices and *P* = .021 for Invsimpson indices). In β‐diversity analysis, there was a minor trend of aggregation between different treatment types at baseline (Figure 2B, PERMANOVA *P* < 0.05). As for the taxonomic abundance, *Firmicutes*, *Bacteroidetes* and *Actinobacteria* were the three most abundant phyla in the chemotherapy group and combined group at baseline (Figure 2C–E). *Proteobacteria* (8.27%) outnumbered *Actinobacteria* (3.89%) in the ICI group. At the order level, *Clostridiales*, *Bacteroidales* and *Lactobacillales* dominated all three treatment groups (Figure S1B–D). *Burkholderiales* was the only order significantly enriched in responders in the immunotherapy group at baseline (*P* = .035).

In addition, changes in microbiome with treatment were also assessed. Metagenomics analysis of dynamic fecal samples revealed that the gut microbiome composition in the three treatment groups changed along with the treatment period (Figure 2C–E, Figure S1B–D). We divided samples into four groups according to the collecting time. T0 was defined as samples were collected before treatment or no more than 3 weeks after treatment. These are the baseline samples. T1 was defined as samples were collected between 21 days and 90 days after treatment; T2 was defined as samples were collected between 90 days and 180 days after treatment; T3 was defined as samples were collected after 180 days post treatment. Regarding the baseline that includes samples within 3 weeks of treatment, we compared the pretreatment samples and those collected within 3 weeks after treatment using PERMANOVA analysis and we found no significant differences in the gut microbiome (PERMANOVA *p* value is 0.627, the beta dispersion *p* value is 0.443). These findings suggest that the inclusion of these additional samples did not introduce significant bias into our analysis or alter our overall conclusions in the part of gut microbiome. The number of patients included in each group was shown in Table S2. Regarding the patients whose treatment duration was less than 12 weeks in Figure 1B, they had already progressed after 12 weeks of treatment and switched to other treatment, so no further samples were collected. The patients included in the sampling were receiving the same treatment, and the time point for stopping sampling was when they terminated their current treatment.

For responders with chemotherapy treatment (Figure 2C), *Bacteroidetes* increased at T1 period, and decreased afterwards. *Firmicutes* decreased at T1 and increased at T2. While for non‐responders with chemotherapy treatment, *Firmicutes* increased at T1. For patients with ICI treatment (Figure 2D), responders showed an increase of *Actinobacteria* and *Proteobacteria* at T1. And non‐responders showed an increase of *Firmicutes* and *Proteobacteria* at T1 and T3. For the combined treatment group (Figure 2E), *Firmicutes* and *Actinobacteria* were increased at T2 for responders, while *Firmicutes* were decreased at T2 for non‐responders.

### 3.3 Gut microbiome associated with therapy response are different among treatment groups

To identify correlations between gut microbiota and the clinical benefits rendered by each treatment, differential abundance analysis of baseline gut microbiome was performed. As the clinical background of the patients is complex, and there are several confounding factors (such as gender, age, BMI, tumour stage, prior medication history) that may influence the gut microbiota composition. The permutational multivariate analysis of variance (PERMANOVA) was first performed to evaluate the effect of confounding factors on microbial communities. The detailed results were shown in Table S3. Taking the confounder of enrolled patients who received various lines of treatment in the ICI group as an example, we conducted a thorough and comprehensive evaluation. The descriptive table summarizing the therapy line of the cohort treated with ICI was listed in Table S4. We performed Fisher's test between the therapy lines and found no significant difference (*p* value = .6126). Additionally, we conducted a PERMANOVA analysis to assess the influence of different therapy lines on the gut microbiome. The results showed that the PERMANOVA P value is 0.782 and the beta dispersion *p* value is 0.855, indicating that the confounding factor of therapy line had no significant effect on the gut microbiota.

**FIGURE 3:**
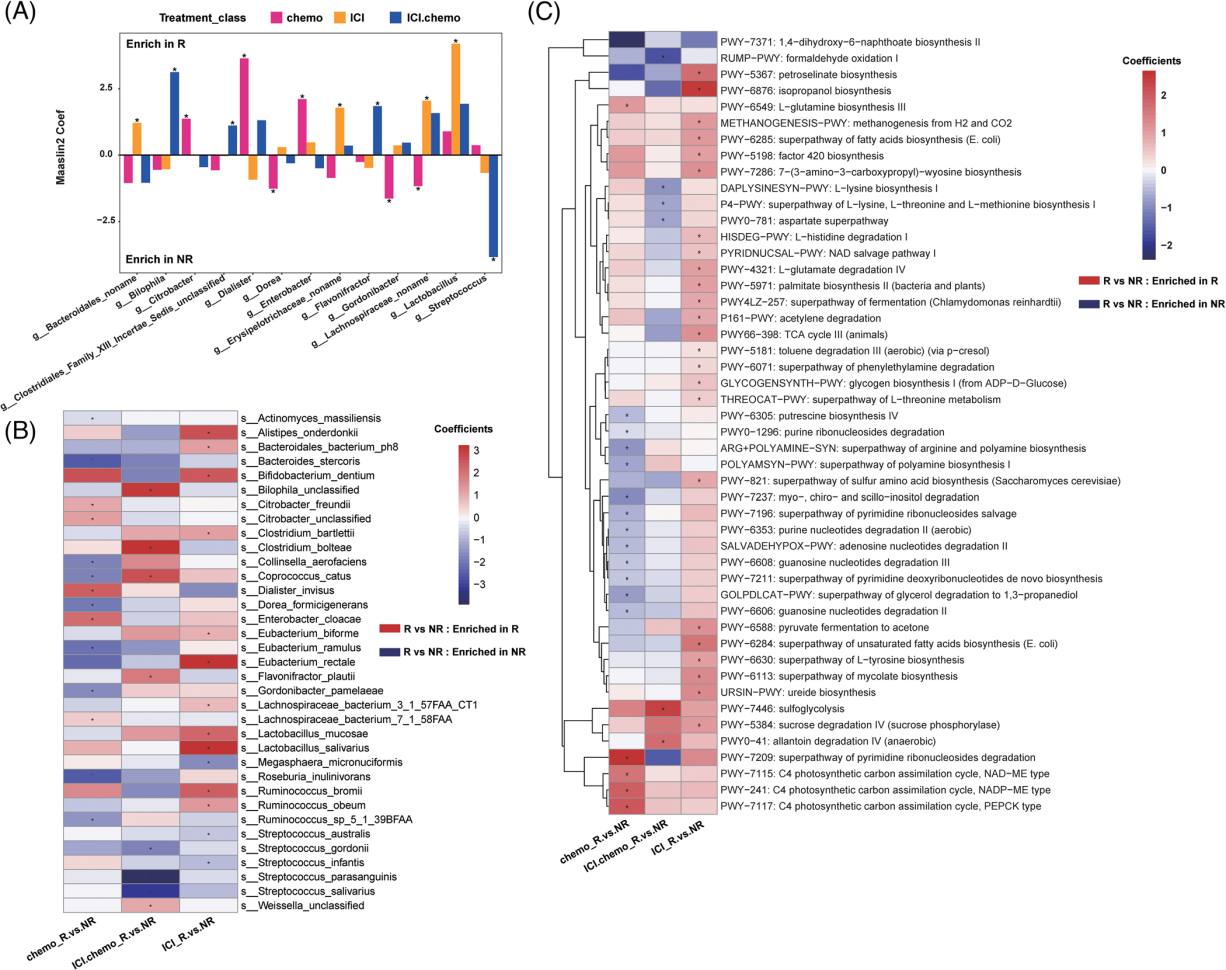
Baseline fecal microbiome associations with cancer treatment and drug response. (A) Bar plots show differentially abundant genera between responders (R) and non‐responders (NR). The y‐axis represents the coefficient value calculated by MaAsLin2 analysis. Taxa exhibiting positive values are enriched in the R group, while taxa exhibiting negative values are enriched in the NR group. Taxa with asterisk represent it is significant different between R and NR group. (B) Heatmap of differentially abundant taxa between R and NR in chemotherapy, ICI‐treatment, and ICI‐combined chemotherapy patients, respectively. (C) Heatmap of differentially abundant metabolic pathways between R and NR in chemotherapy, ICI‐treatment, and ICI‐combined chemotherapy patients, respectively. Asterisks denote statistically significant differences (*p*‐value < .05), based on MaAsLin2 results. The colour of each rectangle represents the coefficient value calculated by MaasLin2 analysis: Red, taxa are enriched in responders, Blue, taxa are enriched in non‐responders.

Significant differences were observed at the genus and species levels (MaAsLin2 P < 0.05). At the genus level, genus *Lactobacillus*, *Erysipelotrichaceae, Ruminooccus* and *Eubacterium* were significantly enriched in Rs of the immunotherapy group, while members of *Bilophila*, *Clostridiales Incertae Sedis XIII*, and *Flavonifractor* were significantly enriched in responders of the combined therapy group. It is noteworthy that the most significant enrichment bacteria in NRs were *Streptococcus* spp. The relative abundances of *Dialister*, *Enterobacter* and *Citrobacter* spp. were significantly enriched in Rs of the chemotherapy group, while members of *Dorea*, *Gordonibacter* and *Lachnospiraceae* were enriched in the NRs. Interestingly, *Lactobacillus* was correlated with favorable responses among all three cohorts. At the species level, in the immunotherapy group, 11 species were enriched in Rs, such as *Lactobacillus mucosae* and *Lactobacillus salivarius*, and three species were reduced. In the immunotherapy plus chemotherapy group, 5 species were enriched in Rs, and three species were reduced, such as *Streptococcus gordonii, Streptococcus parasangunis* and *Streptococcus salivarius*. In the chemotherapy group, five species were enriched in Rs, and eight species were reduced (Figure 3A,B). Additionally, we examined the variation trends of these bacteria in the validation cohort. For patients receiving ICI treatment, we also observed the increased level of *Lactobacillus, L. mucosae* and *L. salivarius* in responders (Figure S2). In the immunotherapy plus chemotherapy group, we observed a trend towards increased abundance of genus *Lactobacillus* and *L. salivarius* in the discovery cohort, which became more pronounced in the validation cohort (MaAsLin2 *P* = .0504 for *Lactobacillus*, MaAsLin2 *P* = .018 for *L. salivarius*) (Figure S2). These findings suggest that gut microbiota, specifically the abundance of *Lactobacillus*, may be associated with the efficacy of ICI and immunotherapy plus chemotherapy treatment in gastric cancer patients.

The differential metabolic pathways between responders and non‐responders at baseline (MaAsLin2 *P* < 0.05) were apparently different among the chemo, ICI and chemo combined ICI group (Figure 3C). As shown in Figure 3C, pathways with red colour in the heatmap represent enrichment in the responder group, while those with blue colour means they are enriched in the non‐responder group. To our surprise, there is no intersection between these three treatment groups. It could be observed that most of the pathways (12/17) are significantly enriched in non‐responders in patients with chemo treatment. Interestingly, most of the NR‐enriched pathways are related to nucleotides metabolism and polyamine metabolism. In contrast, almost all the pathways enriched in responders with ICI treatment are mainly involved in amino acids metabolism, energy metabolism and unsaturated fatty acids metabolism. For those with combined therapy, only two pathways (PWY−7446: sulfoglycolysis and PWY0−41: allantoin degradation IV (anaerobic)) are significantly enriched in R group. And four pathways are significantly enriched in the NR group: RUMP−PWY: formaldehyde oxidation I, DAPLYSINESYN−PWY: L−lysine biosynthesis I, P4−PWY: superpathway of L−lysine, L−threonine and L−methionine biosynthesis I and PWY0−781: aspartate superpathway. These results indicated that the metabolic profile of gut microbiome related to response in the baseline samples is clearly different among various treatments.

### 3.4 The gut microbiome is associated with progression free survival across treatment groups

**FIGURE 4:**
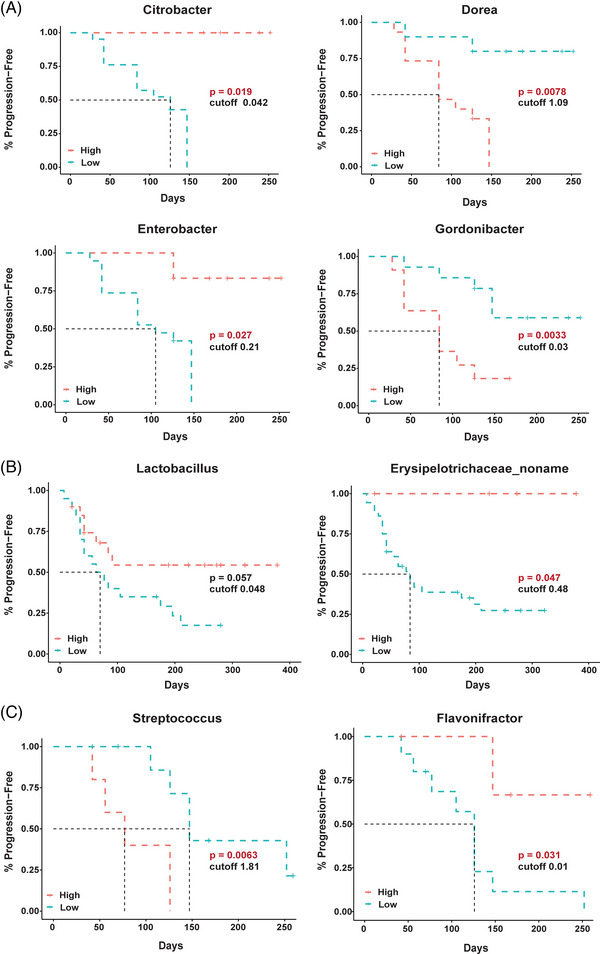
Disparate taxa associated with the survival benefit of chemotherapy (A), ICI‐treatment (B) and ICI‐combined chemotherapy (C) in patients with gastric cancers. Shown are the results of Kaplan–Meier analyses with log‐rank tests, which estimate the median progression‐free survival. The horizontal axis indicates the follow‐up time (days) and the vertical axis represent the percentage of progression‐free survival (%).

Next, we evaluated the relationship between baseline gut microbiota profiles and survival rate at genus and species level. At the genus level, *Citrobacter* (*P* = .019) and *Enterobacter* (*P* = .027) exhibited the strongest positive correlations with PFS, while *Dorea* (*P* = .0078) and *Gordonibacter* (*P* = .0033) presented the strongest negative correlations with PFS in chemotherapy patients (Figure 4A). *Lactobacillus* (*P* = .057) and *Erysipelotrichaceae* (*P* = .047) correlated positively with PFS in immunotherapy patients (Figure 4B). Patients receiving immunotherapy and chemotherapy who harbored depleted *Streptococcus* (*P* = .0063) and elevated *Flavonifractor spp*. (*P* = .031) exhibited superior PFS (Figure 4C). While examining the validation set (Figure S3), we made a fascinating discovery regarding the positively correlation between genus *Lactobacillus* and PFS during ICI therapy (*P* = .024). Furthermore, we also observed a significant association between *Lactobacillus* and PFS in ICI and chemotherapy combination therapy (*P* = .03). These findings highlight the potential benefits of incorporating *Lactobacillus* into cancer treatment protocols.

Additional disparities were observed at the species level (Figure S4). Bacterial species associating positively with PFS in chemotherapy patients included *Citrobacter* unclassified spp. (*P* = .02), *Dialister invisus* (*P* = .024) and *Enterobacter cloacae* (*P* = .028). Bacterial species that was negatively associated with PFS in these patients including *Actionomyces masiliensis* (*P* = .004), *Bacteroides stercoris* (*P* = .019), *Rumminococcus sp 5_1_39BFAA* (*P* = .038), *Gordonibacter pamelaeae* (*P* = .0034), *Eubacterium ramulus* (*P* = .00047), *Coprococcus catus* (*P* = .026) and *Dorea formicigenerans* (*P* = .05). Bacterial species associating positively with PFS in immunotherapy patients included *Alistipes onderdonkii* (*P* = .02), *Eubactrium rectale* (*P* = .0052), *L. salivarius* (*P* = .038) and *L. mucosae* (*P* = .01), while *Streptococcus infantis* (*P* = .02) correlated negatively with PFS in these patients. Only *Flavonifractor plautii* (*P* = .031) was positively associated with PFS in patients receiving both immunotherapy and chemotherapy. In the validation set (Figure S3), we have once again found *L. salivarius* was positively associated with PFS in patients with immunotherapy (*P* = .035) and those with the combined therapy (*P* = .011). These findings provide further evidence of the potential benefits of *Lactobacillus* in cancer treatment and suggest that *L. salivarius* may be a particularly promising candidate to explore in future research.

### 3.5 Characteristics of patients with higher abundance of *Lactobacillus*

**FIGURE 5:**
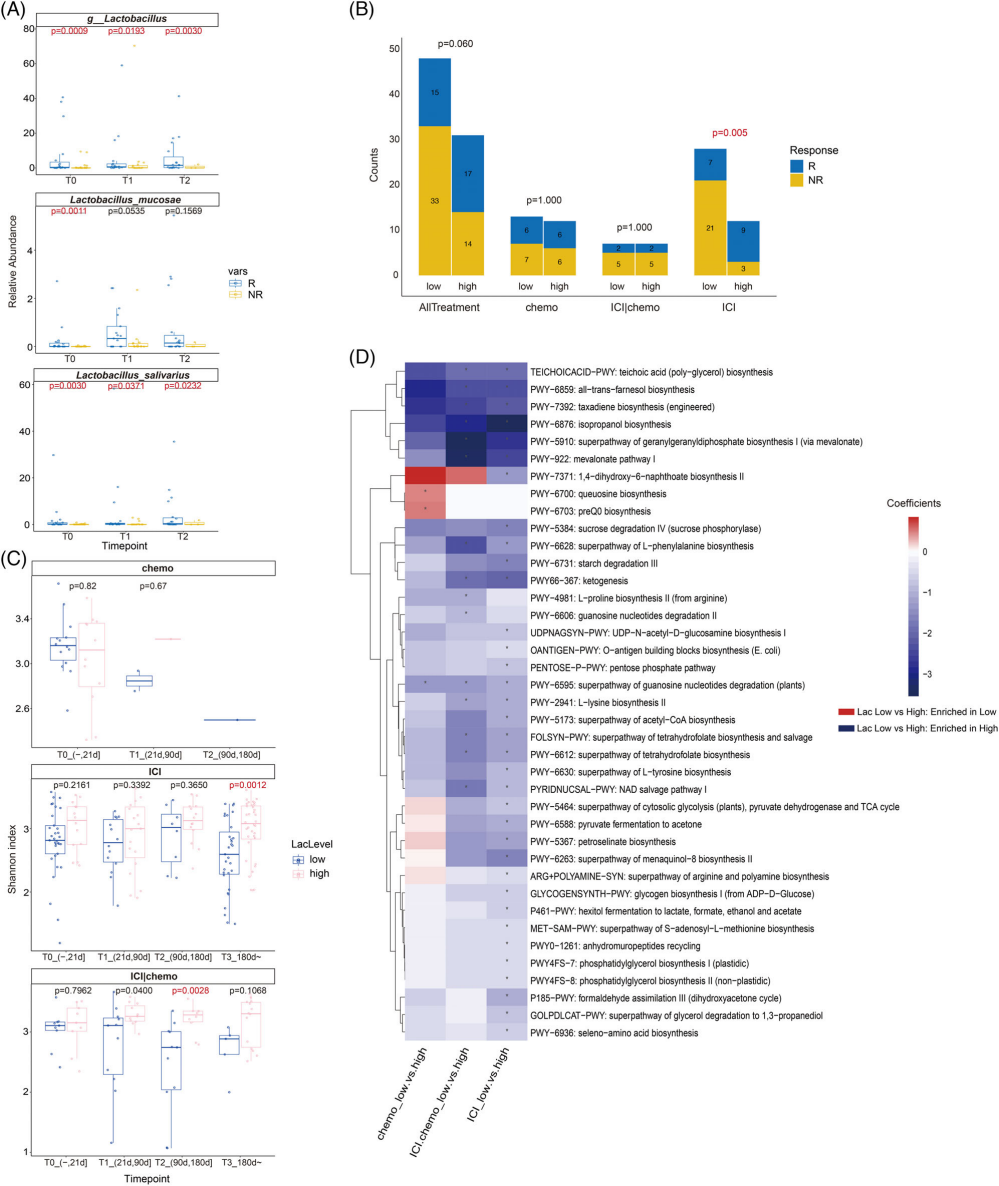
Characteristics of patients with higher abundance of *Lactobacillus*. (A) Relative abundance of genus *Lactobacillus, Lactobacillus mucosae* and *Lactobacillus salivarius* in the ICI group at different timepoints. Comparisons were performed using MaAsLin2. T0 = 0∼21 days, T1 = 21∼90 days, T2 = 90∼days. (B) Number of responders and non‐responders in Lac‐High and Lac‐Low group for each treatment group. Comparisons were performed using Fisher's exact test. (C) Gut microbiome alpha diversity comparison at different timepoints between Lac‐low and Lac‐high patients using Wilcoxon test. (D) Heatmap of differentially bacteria functional pathways between Lac‐high and Lac‐low patients of chemotherapy, immunotherapy, and immunotherapy plus chemotherapy groups. Asterisks denote significantly different (MaAsLin2 coefficient FDR‐adjusted *p* value < .01). The colour of each rectangle represents the coefficient value calculated by MaAsLin2 analysis. Red, pathways enriched in Lac‐low group; blue, pathways enriched in the Lac‐high group. Lac‐High group: patients with elevated abundances of *Lactobacillus* spp. (i.e., greater than the median value for). Lac‐low group: patients with depleted abundances of *Lactobacillus* (i.e., lower than the median value for all samples).

To identify bacteria associated with therapeutic effects, we compared differential microorganisms among the three treatment groups. It is interesting that *Lactobacillus* was consistently enriched in Rs among all treatment groups at baseline. And, it was significantly higher in the immunotherapy group (Figure 3A). In addition, the relative abundance of *Lactobacillus*, and two species *L. mucosae* and *L. salivarius* were consistently enriched in Rs throughout the treatment period in ICI group (Figure 5A).

To further investigate the characteristics of patients with higher abundance of *Lactobacillus*, we divided all 79 patients who had baseline samples into *Lactobacillus* high (Lac‐high) and *Lactobacillus* low (Lac‐low) groups based on the median abundance of *Lactobacillus* at baseline. It is noteworthy that patients with higher *Lactobacillus* were more likely to be responders (*P* = .06), and it was significant in the ICI group (*P* = .005) (Figure 5B). Then we investigated the microbiome characteristics of these patients. We observed a significantly higher microbial diversity in the Lac‐high group who underwent immunotherapy or combined therapy (Figure 5C). We further explored the characteristics of bacterial functional pathways in patients with higher abundance of *Lactobacillus* among the three treatment groups. Interestingly, similar tendency was observed in immunotherapy and the combined therapy group, while the tendency was significantly different in the chemotherapy group. In total 39 metabolic pathways were significantly different between Lac‐low and Lac‐high groups. Two pathways were enriched in Lac‐low patients with chemotherapy, while 30 and 29 pathways were enriched in Lac‐high patients with immunotherapy and immunotherapy plus chemotherapy (Figure 5D).

## 4 DISCUSSION

In our study, we aimed to investigate the relationship between gut microbiota and treatment response in cancer patients. To achieve this, we analyzed the microbial characteristics of responders and non‐responders for each treatment modality, including chemotherapy, immunotherapy, and ICI plus chemotherapy. We also explored the temporal changes of these microbiota over the course of treatment. Furthermore, we examined the correlation between these microbes and PFS, a key clinical outcome for cancer patients.

Of the microbial taxa enriched in responders of ICI monotherapy group, we observed that *L. mucosae* and *L. salivarius* were related to favorable response in immunotherapy patients. There was a noticeable correlation between the PFS benefit and the abundance of *Lactobacillus*, which was further validated in the external validation cohort. Corroborating this association, time‐series results showed the abundance of *Lactobacillus*, especially *L. mucosae* was constantly enriched in responders and depleted in non‐responders. In our previous study, *Lactobacillus* was shown to be associated with anti‐PD‐1/PD‐L1 therapeutic benefits in GI pan‐cancers.^18^ In a similar vein, higher abundances of *Lactobacillus* in responders were observed in immunotherapy cohorts of melanoma^25^ and non‐small cell lung cancer patients.^26^ Taken together with our results in gastric cancer, *Lactobacillus* appears to synergistically enhance and/or promote the effects of immunotherapy in pan‐cancers. The mechanisms by which *Lactobacillus* confer benefits to immunotherapy are seemingly complex and yet to be understood. Previous studies on *Lactobacillus rhamnosus GG* (LGG^27^, ^28^, ^29^;) showed its immune activating effects through the induction of cGAS/STING‐dependent type‐I interferon.^30^ Other studies on *Lactobacillus acidophilus 606* showed its anti‐cancer effect through the secretion of soluble polysaccharides.^31^ Because there are great differences among different *Lactobacillus* species and even strains,^32^ additional research is required to elucidate these mechanisms on the two specific species. Moreover, we observed patients with high relative abundance of *Lactobacillus* presented higher alpha‐diversity and tended to benefit from immunotherapy. Thus, *Lactobacillus* may help to boost the immunotherapy efficacy indirectly by mediating a more diverse gut microenvironment. In chemotherapy plus ICI group, *Lactobacillus* was also found to be significantly enriched in responders in the external validation cohort, patients with higher *Lactobacillus* abundance also possessed higher alpha‐diversity. Higher alpha‐diversity was previously reported to be positively associated with better response in patients with malignant melanoma^15^ and non‐small cell lung cancer.^26^ Aside from *Lactobacillus*, other microbes related to immunotherapy were also identified, such as *R. bromi* and *E. rectale*. Previous study found that castalagin can bind to *R. bromii* and promoted an anticancer response.^33^ *E. rectale* was reported to be associated with increased butyrate secretion,^34^ and butyrate may boost anti‐tumour effect in an ID2‐dependent manner by promoting the IL‐12 signaling pathway.^35^ Whether these species play a similar role in gastric cancer remains to be verified.

The combination of ICI and traditional anti‐tumour regimens such as chemotherapy are common strategy in clinical practice, and it is still unclear whether these combined strategies have synergistic anti‐tumour effects or just a simple superposition. In our cohort, we found that patients receiving ICI plus chemotherapy have a distinct response‐associated microbiome signature from either ICI or chemotherapy alone. Specifically, we found that the abundance of *Streptococcus* in responders was significantly lower than that of non‐responders. Abundance disparities between *S. salivaius* and *S. parasanguinis* were the most significant, and PFS of patients harboring elevated abundances of *Streptococcus* spp. was significantly abbreviated. *Streptococcus* alteration has previously been linked to gastric cancer and liver metastasis.^36^ With respect to gastric cancer, *Streptococcus* has been shown to be associated with the emergence and persistence of atrophy and intestinal metaplasia.^37^ Targeted analysis on tumour suppressor genes revealed that the presence of *Streptococcus* spp. correlated significantly with MLH1 promoter hypermethylation, which promoted progression of the tumour.^38^ Taken together, the high quantities of *Streptococcus* may not only promote the occurrence of gastric cancer but also impact the cancer treatment outcome, which could be potential biomarkers for cancer diagnosis and/or patient stratification.

Our study's novelty lies in investigating the relationship between gut microbiota and the efficacy of different gastric cancer treatment modalities, which has not been extensively explored before. Although chemotherapy was previously the primary treatment for gastric cancer, recent studies have demonstrated that some patients can benefit from immunotherapy alone, as seen in Keynote‐062 and CheckMate‐649.^14^ Furthermore, the combination of immunotherapy and chemotherapy has become the mainstream first‐line treatment for gastric cancer. However, it remains unclear whether there is a unique synergistic effect between immunotherapy and chemotherapy. By conducting a comprehensive comparison of three different treatment methods, our study evaluates whether the gut microbiota associated with combined treatment regimens overlap with those related to immunotherapy or chemotherapy alone. This analysis may help elucidate the unique mode of action of combined treatment in exerting anti‐tumour effects from the perspective of gut microbiota.

With further validation of these findings in an external validation cohort, this study offers a comprehensive set of profiles that link the efficacy of three primary therapeutic regimens for gastric cancer to the differential abundance of specific bacterial taxa in the gut microbiomes of patients. Our study showed gut microbiome might be a potential predictive biomarker for response of common clinical gastric cancer treatment regimens and provided here a solid foundation for emerging adjuvant treatment of fecal microbiota transplantation. Although these findings provide valuable insights, further in‐depth mechanistic exploration is still required to fully understand the underlying biological mechanisms and establish causality.

## 5 CONCLUSION

Overall, our study provides important insights into the relationship between gut microbiota and treatment response in cancer patients. Our findings suggest that *Lactobacillus* may be a key player in this relationship, and that targeting *Lactobacillus* and other beneficial bacteria may hold promise for improving treatment outcomes in cancer patients. These results also highlight the importance of considering the gut microbiome in cancer treatment and management and suggest that microbiome‐based interventions may be a promising avenue for improving patient outcomes.

## CONFLICT OF INTEREST STATEMENT

The authors declare no conflict of interest.

## Supporting information


**Figure S1. Gut microbiome composition associated with treatment and drug response. (A)** Gut microbiome alpha diversity between different treatments (“chemo” for chemotherapy, “ICI” for immune checkpoint inhibitor monotherapy and “ICI|chemo” for chemotherapy plus immune checkpoint inhibitor) at baseline for responders and non‐responders, respectively (Wilcoxon test). **(B)** Dynamic microbial composition of Rs and NRs in chemotherapy group at order level. **(C)** Dynamic microbial composition of Rs and NRs in ICI group at order level. **(D)** Dynamic microbial composition of Rs and NRs in ICI plus chemotherapy group at order level.

**Figure S2**. For patients receiving ICI treatment and immunotherapy plus chemotherapy treatment (ICI_chemo), abundance of *Lactobacillus*, *L. mucosae* and *L. salivarius* in responders and non‐responders in the discovery and validation cohort were ploted.

**Figure S3. Disparate taxa associated with the survival benefit of ICI‐treatment (A and B) and ICI‐combined chemotherapy (C and D) in patients with gastric cancers in the validation cohort**. Shown are the results of Kaplan–Meier analyses with log‐rank tests, which estimate the median progression‐free survival. The horizontal axis indicates the follow‐up time (days) and the vertical axis represent the percentage of progression‐free survival (%).

**Figure S4. Disparate taxa (species level) associated with the survival benefit of chemotherapy (A), ICI‐treatment (B) and ICI‐combined chemotherapy (C) in patients with gastric cancers in the discovery cohort**. Shown are the results of Kaplan–Meier analyses with log‐rank tests, which estimate the median progression‐free survival. The horizontal axis indicates the follow‐up time (days) and the vertical axis represent the percentage of progression‐free survival (%).

Supporting infromation

Supporting infromation

Supporting infromation

Supporting infromation

## Data Availability

Data are available upon reasonable request.

## References

[1] Al‐Batran S‐E , Hartmann JT , Probst S , et al. Phase III trial in metastatic gastroesophageal adenocarcinoma with fluorouracil, leucovorin plus either oxaliplatin or cisplatin: a study of the Arbeitsgemeinschaft Internistische Onkologie. J Clin Oncol. 2008;26(9):1435‐1442. 18349393 10.1200/JCO.2007.13.9378

[2] Bouche O , Raoul JL , Bonnetain F , et al. Randomized multicenter phase II trial of a biweekly regimen of fluorouracil and leucovorin (LV5FU2), LV5FU2 plus cisplatin, or LV5FU2 plus irinotecan in patients with previously untreated metastatic gastric cancer: a Federation Francophone de Cancerologie Digestive Group Study–FFCD 9803. J Clin Oncol. 2004;22(21):4319‐4328. 15514373 10.1200/JCO.2004.01.140

[3] Kim GM , Jeung H‐C , Rha SY , et al. A randomized phase II trial of S‐1‐oxaliplatin versus capecitabine‐oxaliplatin in advanced gastric cancer. Eur J Cancer. 2012;48(4):518‐526. 22243774 10.1016/j.ejca.2011.12.017

[4] Kang Y‐K , Kang W‐K , Shin D‐B , et al. Capecitabine/cisplatin versus 5‐fluorouracil/cisplatin as first‐line therapy in patients with advanced gastric cancer: a randomised phase III noninferiority trial. Ann Oncol. 2009;20(4):666‐673. 19153121 10.1093/annonc/mdn717

[5] Ajani JA , Fodor MB , Tjulandin SA , et al. Phase II multi‐institutional randomized trial of docetaxel plus cisplatin with or without fluorouracil in patients with untreated, advanced gastric, or gastroesophageal adenocarcinoma. J Clin Oncol. 2005;23(24):5660‐5667. 16110025 10.1200/JCO.2005.17.376

[6] Gadgeel SM , Shields AF , Heilbrun LK , et al. Phase II study of paclitaxel and carboplatin in patients with advanced gastric cancer. Am J Clin Oncol. 2003;26(1):37‐41. 12576922 10.1097/00000421-200302000-00008

[7] Ilson DH , Wadleigh RG , Leichman LP , Kelsen DP . Paclitaxel given by a weekly 1‐h infusion in advanced esophageal cancer. Ann Oncol. 2007;18(5):898‐902. 17351256 10.1093/annonc/mdm004

[8] Fuchs CS , Doi T , Jang RW , et al. Safety and efficacy of pembrolizumab monotherapy in patients with previously treated advanced gastric and gastroesophageal junction cancer: phase 2 clinical KEYNOTE‐059 trial. JAMA Oncol. 2018;4(5):e180013. 29543932 10.1001/jamaoncol.2018.0013 PMC5885175

[9] Kang Y‐K , Boku N , Satoh T , et al. Nivolumab in patients with advanced gastric or gastro‐oesophageal junction cancer refractory to, or intolerant of, at least two previous chemotherapy regimens (ONO‐4538‐12, ATTRACTION‐2): a randomised, double‐blind, placebo‐controlled, phase 3 trial. Lancet. 2017;390(10111):2461‐2471. 28993052 10.1016/S0140-6736(17)31827-5

[10] Chao J , Fuchs CS , Shitara K , et al. Pembrolizumab (pembro) in microsatellite instability‐high (MSI‐H) advanced gastric/gastroesophageal junction (G/GEJ) cancer by line of therapy. J Clin Oncol. 2020;38(4_suppl):430‐430.

[11] Li J , Deng Y , Zhang W , et al. Subcutaneous envafolimab monotherapy in patients with advanced defective mismatch repair/microsatellite instability high solid tumors. J Hematol Oncol. 2021;14(1):95. 34154614 10.1186/s13045-021-01095-1 PMC8218452

[12] Kim ST , Cristescu R , Bass AJ , et al. Comprehensive molecular characterization of clinical responses to PD‐1 inhibition in metastatic gastric cancer. Nat Med. 2018;24(9):1449‐1458. 30013197 10.1038/s41591-018-0101-z

[13] Xie T , Liu Y , Zhang Z , et al. Positive status of Epstein‐Barr virus as a biomarker for gastric cancer immunotherapy: a prospective observational study. J Immunother. 2020;43(4):139‐144. 32134806 10.1097/CJI.0000000000000316 PMC7144749

[14] Janjigian YY , Shitara K , Moehler M , et al. First‐line nivolumab plus chemotherapy versus chemotherapy alone for advanced gastric, gastro‐oesophageal junction, and oesophageal adenocarcinoma (CheckMate 649): a randomised, open‐label, phase 3 trial. Lancet. 2021;398(10294):27‐40. 34102137 10.1016/S0140-6736(21)00797-2 PMC8436782

[15] Gopalakrishnan V , Spencer CN , Nezi L , et al. Gut microbiome modulates response to anti‐PD‐1 immunotherapy in melanoma patients. Science. 2018;359(6371):97‐103. 29097493 10.1126/science.aan4236 PMC5827966

[16] Yi Y , Shen L , Shi W , et al. Gut microbiome components predict response to neoadjuvant chemoradiotherapy in patients with locally advanced rectal cancer: a prospective, longitudinal study. Clin Cancer Res. 2021;27(5):1329‐1340. 33298472 10.1158/1078-0432.CCR-20-3445

[17] Yu T , Guo F , Yu Y , et al. Fusobacterium nucleatum promotes chemoresistance to colorectal cancer by modulating autophagy. Cell. 2017;170(3):548‐563.e16. 28753429 10.1016/j.cell.2017.07.008 PMC5767127

[18] Peng Z , Cheng S , Kou Y , et al. The gut microbiome is associated with clinical response to anti‐PD‐1/PD‐L1 immunotherapy in gastrointestinal cancer. Cancer Immunol Res. 2020;8(10):1251‐1261. 32855157 10.1158/2326-6066.CIR-19-1014

[19] Eisenhauer EA , Therasse P , Bogaerts J , et al. New response evaluation criteria in solid tumours: revised RECIST guideline (version 1.1). Eur J Cancer. 2009;45(2):228. 19097774 10.1016/j.ejca.2008.10.026

[20] Kluger HM , Tawbi HA , Ascierto ML , et al. Defining tumor resistance to PD‐1 pathway blockade: recommendations from the first meeting of the SITC Immunotherapy Resistance Taskforce. J Immunother Cancer. 2020;8(1):e000398. 32238470 10.1136/jitc-2019-000398 PMC7174063

[21] Bolger AM , Lohse M , Usadel B . Trimmomatic: a flexible trimmer for illumina sequence data. Bioinformatics. 2014;30(15):2114‐2120. 24695404 10.1093/bioinformatics/btu170 PMC4103590

[22] Franzosa EA , Mciver LJ , Rahnavard G , et al. Species‐level functional profiling of metagenomes and metatranscriptomes. Nat Methods. 2018;15(11):962‐968. 30377376 10.1038/s41592-018-0176-y PMC6235447

[23] Zhu Z , Satten GA , Mitchell C , Hu Y‐J . Constraining PERMANOVA and LDM to within‐set comparisons by projection improves the efficiency of analyses of matched sets of microbiome data. Microbiome. 2021;9(1):133. 34108046 10.1186/s40168-021-01034-9 PMC8191060

[24] Mallick H , Rahnavard A , Mciver LJ , et al. Multivariable association discovery in population‐scale meta‐omics studies. PLoS Comput Biol. 2021;17(11):e1009442. 34784344 10.1371/journal.pcbi.1009442 PMC8714082

[25] Matson V , Fessler J , Bao R , et al. The commensal microbiome is associated with anti‐PD‐1 efficacy in metastatic melanoma patients. Science. 2018;359(6371):104‐108. 29302014 10.1126/science.aao3290 PMC6707353

[26] Katayama Y , Yamada T , Shimamoto T , et al. The role of the gut microbiome on the efficacy of immune checkpoint inhibitors in Japanese responder patients with advanced non‐small cell lung cancer. Transl Lung Cancer Res. 2019;8(6):847‐853. 32010563 10.21037/tlcr.2019.10.23 PMC6976345

[27] Fang S‐B , Shih H‐Y , Huang C‐H , Li L‐T , Chen C‐C , Fang H‐W . Live and heat‐killed Lactobacillus rhamnosus GG upregulate gene expression of pro‐inflammatory cytokines in 5‐fluorouracil‐pretreated Caco‐2 cells. Support Care Cancer. 2014;22(6):1647‐1654. 24500789 10.1007/s00520-014-2137-z

[28] Riehl TE , Alvarado D , Ee X , et al. Lactobacillus rhamnosus GG protects the intestinal epithelium from radiation injury through release of lipoteichoic acid, macrophage activation and the migration of mesenchymal stem cells. Gut. 2019;68(6):1003‐1013. 29934438 10.1136/gutjnl-2018-316226 PMC7202371

[29] Cai S , Kandasamy M , Rahmat JN , et al. Lactobacillus rhamnosus GG activation of dendritic cells and neutrophils depends on the dose and time of exposure. J Immunol Res. 2016;2016: 7402760. 27525288 10.1155/2016/7402760 PMC4971325

[30] Wei Si , Hua L , Bugno J , et al. Lactobacillus rhamnosus GG induces cGAS/STING‐ dependent type I interferon and improves response to immune checkpoint blockade. Gut. 2022;71:521‐533. 33685966 10.1136/gutjnl-2020-323426 PMC8710942

[31] Choi SS , Kim Y , Han KS , You S , Oh S , Kim SH . Effects of Lactobacillus strains on cancer cell proliferation and oxidative stress in vitro. Lett Appl Microbiol. 2006;42(5):452‐458. 16620202 10.1111/j.1472-765X.2006.01913.x

[32] Mcfarland LV , Evans CT , Goldstein EJC . Strain‐specificity and disease‐specificity of probiotic efficacy: a systematic review and meta‐analysis. Front Med (Lausanne). 2018;5:124. 29868585 10.3389/fmed.2018.00124 PMC5949321

[33] Messaoudene M , Pidgeon R , Richard C , et al. A natural polyphenol exerts antitumor activity and circumvents anti‐PD‐1 resistance through effects on the gut microbiota. Cancer Discov. 2022;12(4):1070‐1087. 35031549 10.1158/2159-8290.CD-21-0808 PMC9394387

[34] Tanaka S , Yamamoto K , Yamada K , Furuya K , Uyeno Y . Relationship of enhanced butyrate production by colonic butyrate‐producing bacteria to immunomodulatory effects in normal mice fed an insoluble fraction of brassica rapa L. Appl Environ Microbiol. 2016;82(9):2693‐2699. 26921420 10.1128/AEM.03343-15 PMC4836433

[35] He Y , Fu L , Li Y , et al. Gut microbial metabolites facilitate anticancer therapy efficacy by modulating cytotoxic CD8(+) T cell immunity. Cell Metab. 2021;33(5):988‐1000.e7. 33761313 10.1016/j.cmet.2021.03.002

[36] Yu D , Yang J , Jin M , et al. Fecal streptococcus alteration is associated with gastric cancer occurrence and liver metastasis. mBio. 2021;12(6):e0299421. 34872346 10.1128/mBio.02994-21 PMC8649758

[37] Sung JJY , Coker OO , Chu E , et al. Gastric microbes associated with gastric inflammation, atrophy and intestinal metaplasia 1 year after Helicobacter pylori eradication. Gut. 2020;69(9):1572‐1581. 31974133 10.1136/gutjnl-2019-319826 PMC7456733

[38] Xia X , Wu WK , Wong SH , et al. Bacteria pathogens drive host colonic epithelial cell promoter hypermethylation of tumor suppressor genes in colorectal cancer. Microbiome. 2020;8(1):108. 32678024 10.1186/s40168-020-00847-4 PMC7367367

