# Supplementary figures and images for: The gut microbiome affects response of treatments in HER2‐negative advanced gastric cancer
Source: Clin Transl Med. 2023 Jun 28;13(7):e1312. doi: 10.1002/ctm2.1312 (PMC10307992; doi:10.1002/ctm2.1312)

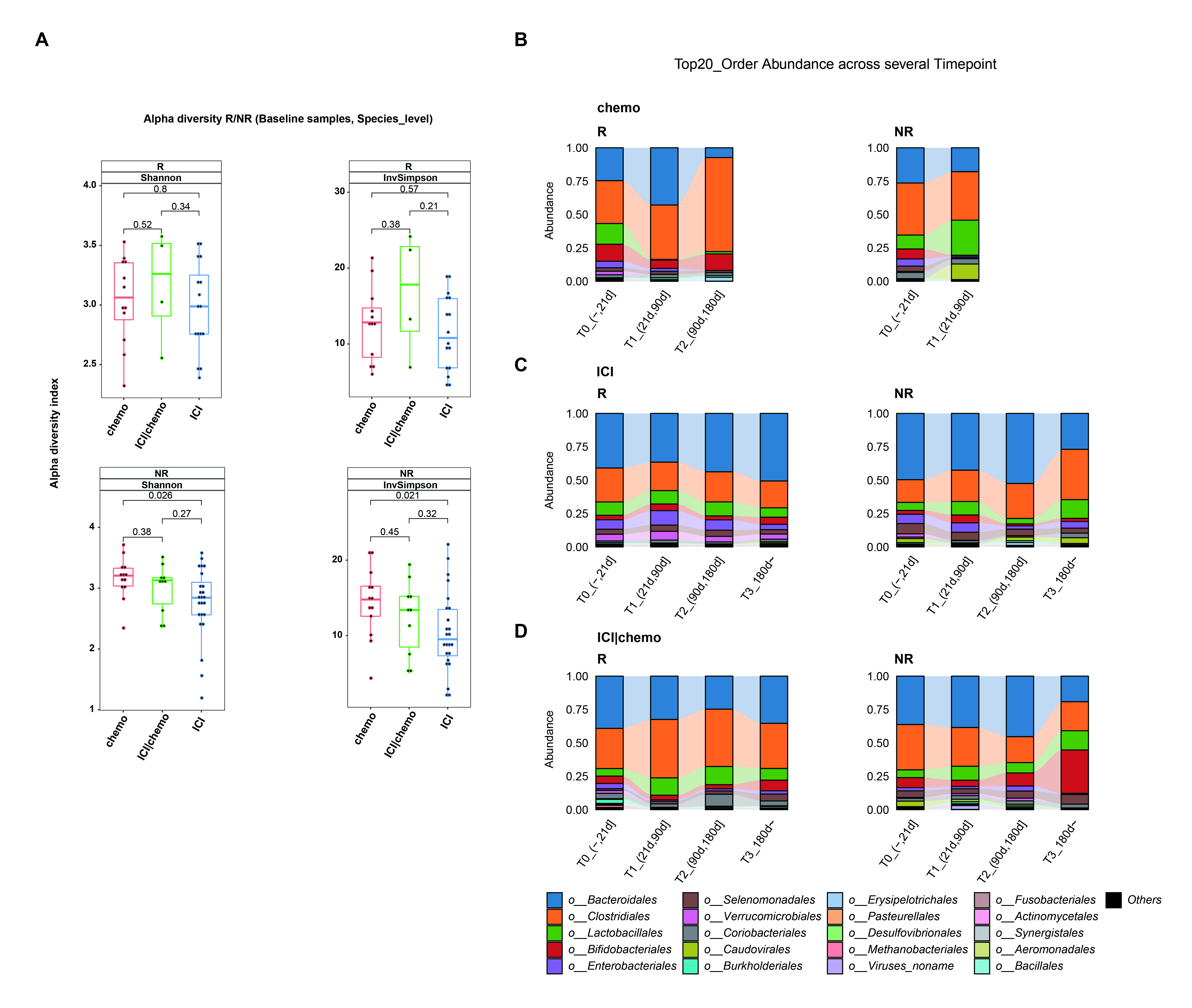

Supplement: Supplementary file 1 — Figure S1. Gut microbiome composition associated with treatment and drug response. (A) Gut microbiome alpha diversity between different treatments (“chemo” for chemotherapy, “ICI” for immune checkpoint inhibitor monotherapy and “ICI|chemo” for chemotherapy plus immune checkpoint inhibitor) at baseline for responders and non‐responders, respectively (Wilcoxon test). (B) Dynamic microbial composition of Rs and NRs in chemotherapy group at order level. (C) Dynamic microbial composition of Rs and NRs in ICI group at order level. (D) Dynamic microbial composition of Rs and NRs in ICI plus chemotherapy group at order level. [file CTM2-13-e1312-s008.tif]

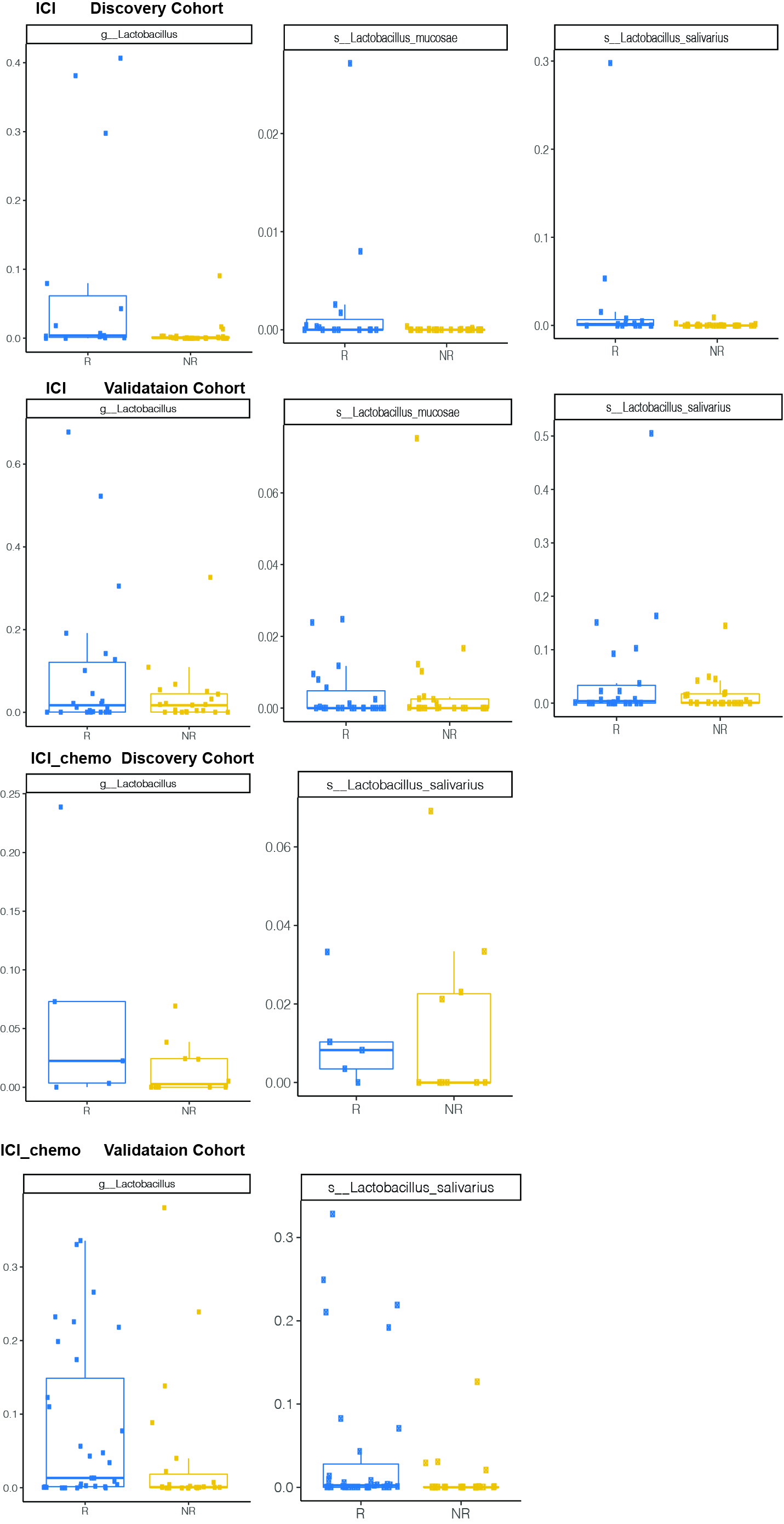

Supplement: Supplementary file 2 — Figure S2. For patients receiving ICI treatment and immunotherapy plus chemotherapy treatment (ICI_chemo), abundance of Lactobacillus, L. mucosae and L. salivarius in responders and non‐responders in the discovery and validation cohort were ploted. [file CTM2-13-e1312-s007.tif]

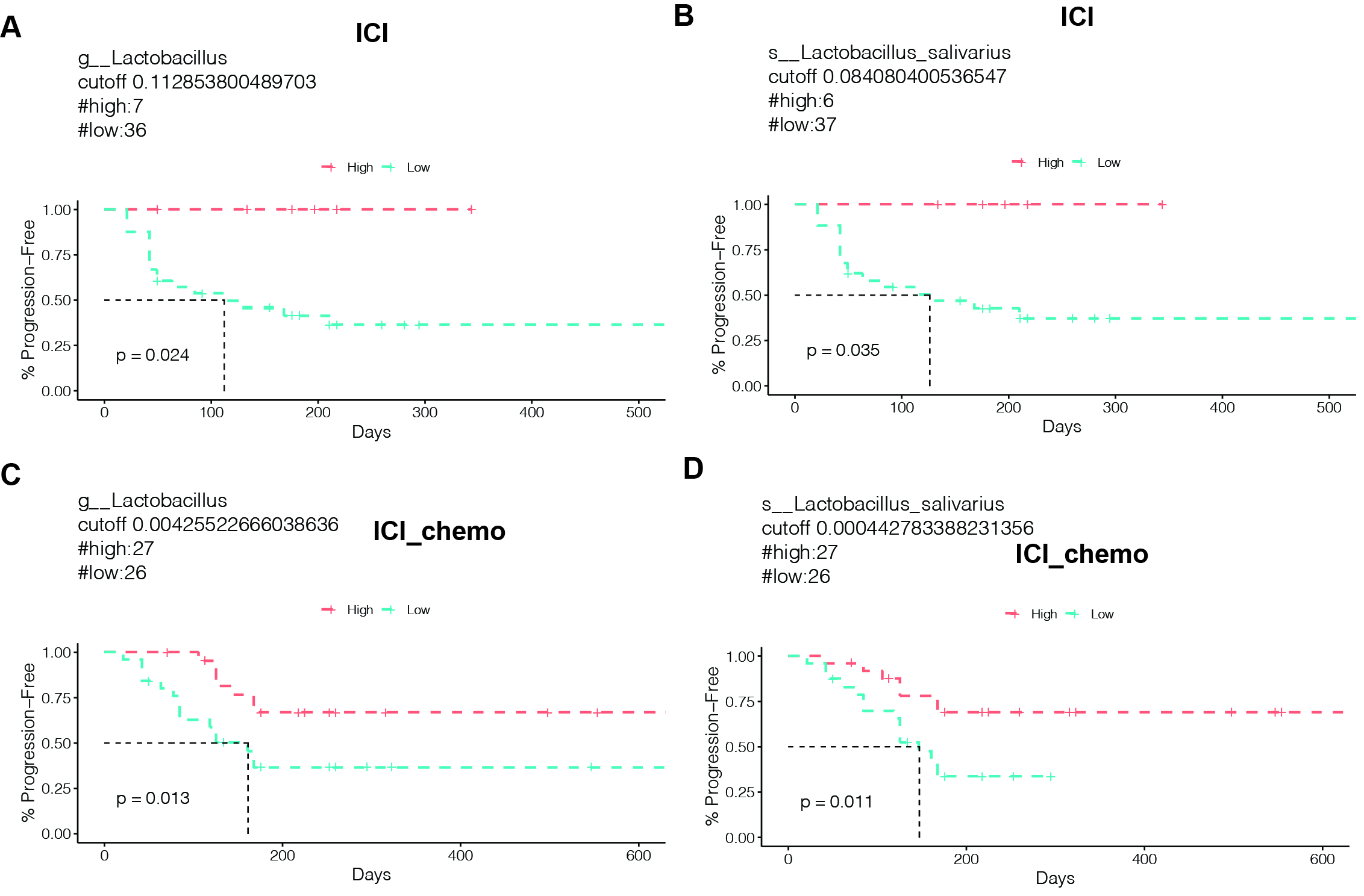

Supplement: Supplementary file 3 — Figure S3. Disparate taxa associated with the survival benefit of ICI‐treatment (A and B) and ICI‐combined chemotherapy (C and D) in patients with gastric cancers in the validation cohort. Shown are the results of Kaplan–Meier analyses with log‐rank tests, which estimate the median progression‐free survival. The horizontal axis indicates the follow‐up time (days) and the vertical axis represent the percentage of progression‐free survival (%). [file CTM2-13-e1312-s005.tif]

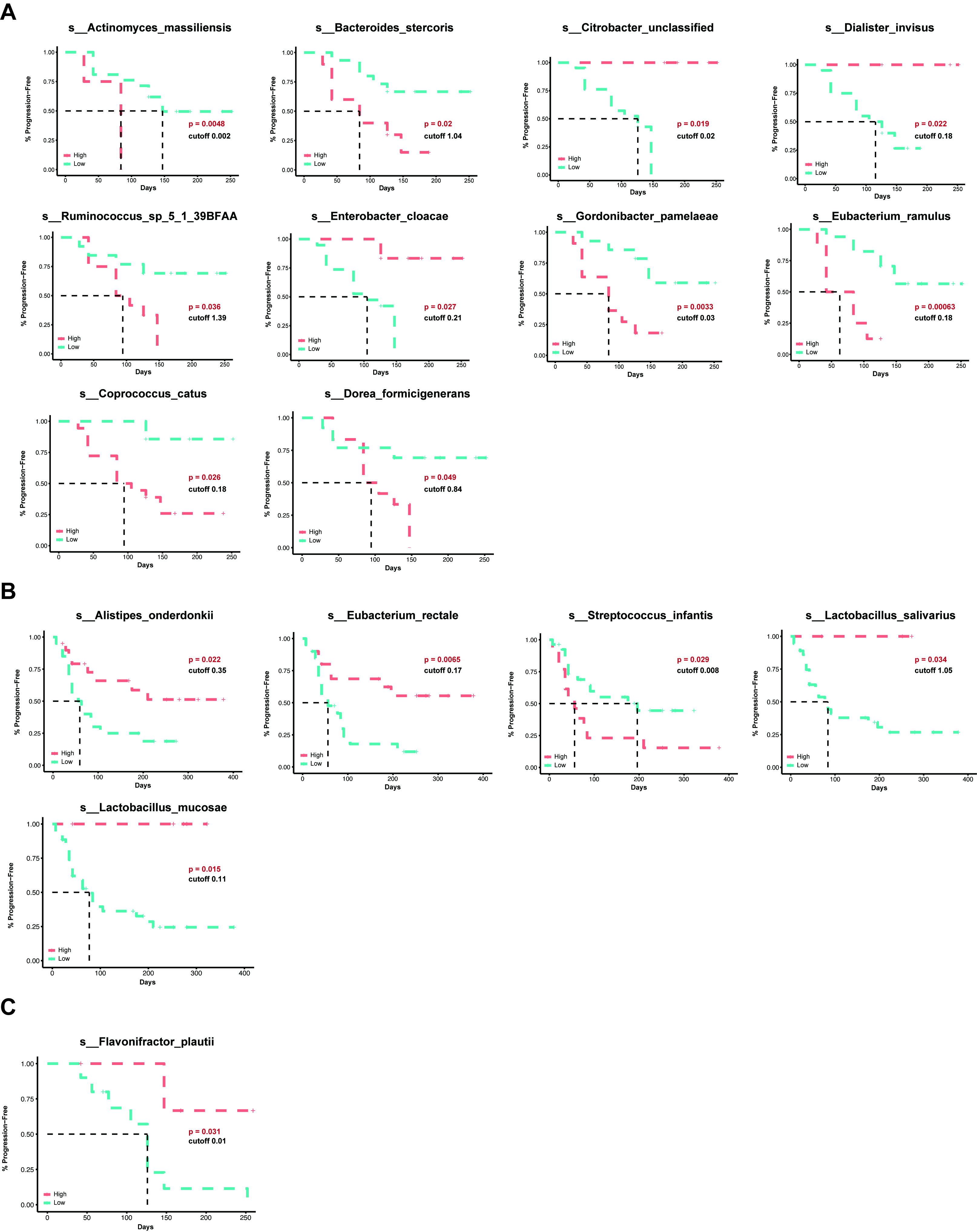

Supplement: Supplementary file 4 — Figure S4. Disparate taxa (species level) associated with the survival benefit of chemotherapy (A), ICI‐treatment (B) and ICI‐combined chemotherapy (C) in patients with gastric cancers in the discovery cohort. Shown are the results of Kaplan–Meier analyses with log‐rank tests, which estimate the median progression‐free survival. The horizontal axis indicates the follow‐up time (days) and the vertical axis represent the percentage of progression‐free survival (%). [file CTM2-13-e1312-s002.tif]
